# Mental health self-stigma moderates the effect of social support on depression, anxiety and stress among young adult Pacific Islanders

**DOI:** 10.1016/j.ssmmh.2025.100471

**Published:** 2025-06-23

**Authors:** Melanie D. Sabado-Liwag, James Russell Pike, Mayra Zamora, Cindy Garcia, Lolofi Soakai, Genesis Lutu, Paula H. Palmer, Sherine El-Toukhy, Patchareeya P. Kwan

**Affiliations:** a California State University, Los Angeles, Department of Public Health, United States; b New York University, Grossman School of Medicine, United States; c Motivating Action Leadership Opportunity (MALO), United States; d Claremont Graduate University, School of Community and Global Health, United States; e National Institutes of Health, National Institute of Minority Health and Health Disparities, United States; f California State University, Northridge, Department of Health Sciences, United States

**Keywords:** Pacific Islanders, Social support, Mental health, Depression, Anxiety, Stress, Stigma

## Abstract

**Objectives:**

In the United States, Pacific Islanders have a high documented prevalence of depression, anxiety, and stress yet report low use of mental health services. Little is known about their risk and protective factors against psychological distress, such as self-stigma and social support. The aim of the current study was to investigate how perceived levels of social support and levels of self-stigma moderate mental health outcomes (depression, anxiety, and stress).

**Methods:**

A cross-sectional study was conducted in Southern California from May 2018 to June 2019 of 213 Pacific Islanders aged 18–35 years who had never been medically diagnosed with a severe mental condition. Each participant completed an online survey that assessed their demographics, mental health, perceptions of mental health, and perceived social support. The independent and synergistic effects of mental health self-stigma and social support on self-reported depression, anxiety, and stress were examined in a series of demographics-adjusted linear regression models.

**Results:**

Social support from family members exhibited a protective effect on all three outcomes. Social support from friends was associated with higher levels of depression, anxiety, and stress. Self-stigma acted as a moderator that either amplified the deleterious effects associated with social support from friends or altered the protective effect of support from family.

**Conclusions:**

Findings suggest that social support from friends may play a role in the presence of self-stigma through peer stigmatization of psychological distress. Family- and peer-centric interventions are needed to reduce self-stigma and improve mental health among young adult Pacific Islanders.

## 1. Introduction

Representing roughly 7 % of the US population, 16.6 % of Pacific Islander adults reported a mental health issue in 2019 (SAMHSA, 2020) and 14 % reported serious psychological distress in the past year (SAMSHA, 2024). Since, surveillance trends indicate increased trauma-related psychological distress and higher risks for suicidality, specifically among Pacific Islander young adults (Reyes et al., 2024), adult males (CDC, 2023), and female adolescents (Office of Minority Health, 2024). Although limited, current mental health literature on Pacific Islanders is growing (Nguyen et al., 2025; Cutrer-Párraga et al., 2024; Subica et al., 2019; Ðoàn et al., 2019) as US federal recognition (since 1997 through the Office of Management and Budget) and local advocacy initiatives distinguish Asian Americans and Pacific Islanders as a two separate racial groups in health research and population surveys, (Cha et al., 2022; Allen et al., 2016; Yamada et al., 2019; Gordon et al., 2019; Morey et al., 2022). Despite comprising roughly 50 subpopulations and over 100 languages (Tanqueco et al., 2020), aggregation of racial/ethnic groups, defaulting ‘other’ or single-race categorization (i.e. unreported multi-ethnic groups) due to limited sample size or lack of initiative to generate differences underestimate the burden of mental illness, masks comorbid chronic health conditions (Yamada et al., 2019; Gordon et al., 2019; Wu and Blazer, 2015; Galinsky et al., 2017), and undermines community-level efforts to improve health disparities in both Asian American and Pacific Islander communities (Sabado-Liwag et al., 2024; Nguyen-Truong et al., 2023; Morey et al., 2022). When appropriate disaggregated discourse are made, reports can show differences and similarities between Asian Americans and Pacific Islanders, both for coalition building and policy/intervention advocacy (Reyes et al., 2024; Tanqueco et al., 2020; Tan et al., 2024). For example, an assessment report by AAPI Data and UCLA Center for Health Policy Research (Tan et al., 2024) provides a granular look into the mental experiences of various subethnic groups. Particularly, Native Hawaiian and Pacific Islanders (24 %) and Asian Americans (16 %) reported needing mental health support during the pandemic, and less than half (31 % and 42 %, respectively) faced mental health access barriers.

From a socio-ecological approach, various factors contribute to poor mental health among Pacific Islanders, which include but are not limited to structural determinants (e.g. colonization and racism, erasure of cultural customs for Western standards, military/political presence, lack of accurate data representation, limited culturally responsive resources or treatment, socioeconomic deprivation), cultural barriers (e.g. mental illness as a weakness/burden; lack of mental health literacy) and interpersonal and intrapersonal determinants (e.g. historical or generational trauma, stigma and stereotypes to mental illness, help-seeking behaviors, familial pressure/expectations) (Goetz et al., 2023; Nguyen et al., 2025; Tanqueco et al., 2020; Eng et al., 2024; Tan et al., 2024). While complex, compounded, and intersectional, these contributors work together to perpetuate psychological distress while hindering mental health services/intervention access and utilization among Pacific Islanders (Nguyen et al., 2025; Kaholokula et al., 2020; Cutrer-Párraga et al., 2024; Kapeli et al., 2020a). Despite evidence of poor Pacific Islander in mental health across the lifespan in the US, they are less likely to receive mental services nor treatment than non-Hispanic Whites (Office of Minority Health, 2024) and Asian Americans (Nguyen et al., 2025).

One crucial component to assessing mental health status is psychological distress, which refers to a range of negative internal stimuli (i.e., depression, anxiety, and stress) that individuals feel in response to external stimuli that are difficult to cope with in daily life (Arvidsdotter et al., 2016). Psychological distress is fluid and can be intensified or alleviated by socioenvironmental, economic, or biological stressors. In the context of socioenvironmental stressors, support from one’s social relationships (i.e., family, friends, community) can act as either a stressor or a buffer to mental health outcomes (Allen et al., 2016; Yamada et al., 2019; Li et al., 2020; Chronister et al., 2013). Similarly, mental health self-stigma may compound psychological distress (Livingston and Boyd, 2010; Carrara and Ventura, 2018; Tsang et al., 2016; Yanos et al., 2015; Lannin et al., 2016; Kaholokula et al., 2020). However, less known about the moderating role of self-stigma on Pacific Islander mental health (Kapeli et al., 2020a; Cutrer-Párraga et al., 2024). To better address documented mental health disparities among Pacific Islanders, we must not only disaggregate their unique communities but also continue understanding the risk and protective factors that operate in this population as well as potential moderators of these factors from a systems-thinking approach (Cutrer-Párraga et al., 2024; Garrett et al., 2025; Kaholokula et al., 2020), which include varying levels and perceptions of social support.

Social support is critical to mental health. Defined here in the context of family, peer, and community networks, social support is the basis for feelings of belonging, acceptance, and community cohesion among individuals (Harandi et al., 2017). Previous research has shown that higher levels of perceived social support encourage usage of mental health services and healing while decreasing levels of self-stigma (Lam and Rosenheck, 1999; Chou and Chronister, 2012; Adewuya et al., 2011). Other studies have determined that social support within various cultural contexts can either positively or negatively influence individual perceptions and attitudes towards mental illness, potentially perpetuating self-stigma and impeding positive mental health outcomes (Allen et al., 2016; Ran et al., 2021). Like many indigenous groups and communities of color, Pacific Islander culture typically favors collectivism and familism impacting health behaviors – such that social relationships are central to one’s identity and admitting to mental distress may be perceived as a disruption to social dynamics and family reputation (Kwan et al., 2023; Garrett et al., 2025; Kalibatseva, 2015; Ran et al., 2021; Kaholokula et al., 2020). Such contrasts illuminate the double-edged sword of social support in mental health outcomes and stigmatization. Social support is viewed as a multi-dimensional construct comprised of distinct networks (i.e., family, friends, significant others), each operating independently of the others (Zimet et al., 1988). Recent research among Chinese adults examined these levels of social support in different health contexts and populations (Khatiwada et al., 2021; Grey et al., 2020). Grey et al. (2020), for example, reported social support from friends, family, and significant others was associated with decreased odds of depression and anxiety during the first year of the COVID-19 pandemic. With a growing mental health literature about social support among Pacific Islanders and given the rising rates of psychological distress due to the pandemic, further analysis on the varying levels of social support in relations to mental health outcomes and moderated by self-stigma among Pacific Islanders is critical to future behavioral health interventions.

Mental health stigma encompasses negative perceptions and social unacceptance towards individuals with a mental illness diagnosis (Vogel et al., 2006, 2007; Adewuya et al., 2011), which can lead to prejudice and discriminatory behaviors (Corrigan, 2004; Ritsher and Phelan, 2004; Subica et al., 2019). External stigma can further harm individuals when it is personally adopted or internalized. The internalization of public stigma is known as self-stigma and occurs when an individual believes and accepts the public’s negative perceptions regarding their condition (Livingston and Boyd, 2010). Self-stigma is shown to have adverse effects on those who are experiencing any mental illness such as negatively affecting an individual’s sense of self and intensifying feelings of hopelessness, self-degradation, self-depreciation, and reduced self-efficacy (Corrigan, 2004, 2006, 2007; Ritsher and Phelan, 2004; Livingston and Boyd, 2010; Adewuya et al., 2011; Fox et al., 2018; Tsang et al., 2016; Vogel et al., 2007) which impedes recovery (Yanos et al., 2015). Furthermore, higher levels of self-stigma are associated with higher levels of poor mental health symptomology, hesitation or avoidance of mental health intervention, and lower levels of treatment adherence as seen among different minority communities (Livingston and Boyd, 2010; Carrara and Ventura, 2018; Tsang et al., 2016; Lannin et al., 2016).

Cultural obligations, collectivist values, religious and spiritual beliefs, misconceptions about mental illness, and generational differences in attitudes towards mental illness have been identified as propagators of mental health self-stigma among Native Hawaiian and Pacific islander populations (Subica et al., 2019; Allen et al., 2016; Yamada et al., 2019; Phan, 2020). When exploring stigma, studies show that Native Hawaiians and Pacific Islanders seldom receive services despite experiencing profound levels of mental health issues (Reyes et al., 2024; Subica et al., 2019; Cutrer-Párraga et al., 2024). Furthermore, the role of self-stigma as a potential moderator on social support remains unexplored in the literature for Pacific Islander mental health. Some recent evidence among Asian subpopulations show strong associations between low self-stigma, support, and psychological distress. For example, among Chinese patients with substance use disorders, higher perceived social support was associated with lower self-stigma (Wang et al., 2022). Another a recent study on internalized stigma related to travel from Hubei Province, China during the first wave of the COVID-19 pandemic found that social support buffered the negative effects of self-stigma on depression and anxiety among travelers (Li et al., 2020). While the directionality of the relationship between self-stigma, social support, and mental health remains unclear, it is possible that self-stigma also modifies the relationship between social support and mental health outcomes, especially for Pacific Islanders.

This study seeks to analyze the relationship between perceived levels of social support (from family, friends, and significant others) and levels of self-stigma on three mental health outcomes (depression, anxiety, and stress) among a non-clinical Pacific Islander sample. It is hypothesized that mental health outcomes are negatively associated with levels of perceived social support, positively associated with levels of self-stigma, and that self-stigma buffers the protective effects of social support on mental health.

## 2. Methods

### 2.1. Sample

Young adult recruitment was leveraged by community partnerships and stakeholder relationships established over a decade from community-based participatory research (CBPR) endeavors through WINCART (Weaving an Islander Network for Cancer Awareness and Research Training (Tanjasiri et al., 2007). As trusted leaders in Pacific Islander-led organizations and social circles, community-based researchers with similar initiatives to understand the mental health of young adult Pacific Islanders were asked to partner in this project not only as trusted health advocates for recruitment, but community representatives for culturally-responsive research methodology and dissemination. From May 2018 to June 2019, 237 Pacific Islanders were recruited throughout Southern California through face-to-face interactions at social events and through phone calls, text messages, and emails with individuals who reached out after hearing about the study from friends and family members. Over 90 % (N = 213) of the Pacific Islanders were classified as eligible and agreed to take part in this expedited study approved by the Institutional Review Board at California State University, Los Angeles. Eligible individuals who (a) self-identified as Pacific Islanders, (b) were between the ages of 18 and 35 years old, and (c) had not been formally diagnosed by a medical professional as having a severe mental condition completed a web-based survey consisting of measures of general health, mental health, mental health stigma, and other health behaviors.

### 2.2. Measures

The short form of the Depression, Anxiety, and Stress Scales (DASS-21) was selected for this study based on prior research indicating the measure exhibited excellent consistency, convergent validity, discriminant validity, and construct validity in multinational populations (Lee et al., 2019; Lee, 2019). The DASS-21 consists of three validated 7-item subscales that measure the severity of past-week mental health symptoms (Lovibond and Lovibond, 1995; Henry and Crawford, 2005). Participants were presented with statements on depression (*“I couldn’t seem to experience any positive feeling at all”*), anxiety (*“I was aware of dryness of my mouth”*), and stress (*“I found myself getting agitated”*), and chose from the following response options for each: 0 = ‘Did not apply to me at all,’ 1 = ‘Applied to me to some degree, or some of the time,’ 2 = ‘Applied to me to a considerable degree, or a good part of time,’ 3 = ‘Applied to me very much, or most of the time.’ Based on published scoring guidelines, the sum of each scale was calculated and multiplied by two (Lovibond and Lovibond, 1995). Each score was then categorized as normal (Depression: 0–9, Anxiety: 0–7, Stress: 0–14), mild (Depression: 10–13, Anxiety: 8–9, Stress: 15–18), moderate (Depression: 14–20, Anxiety: 10–14, Stress: 19–25), severe (Depression: 21–27, Anxiety: 15–19, Stress: 26–33), or extremely severe (Depression: 28+, Anxiety: 20+, Stress: 34+). In the current population, the measure demonstrated excellent internal consistency for depression (α = 0.93) and stress (α = 0.91), and good internal consistency for anxiety (α = 0.88). Similar reliability and consistency was found among Pacific Islander young adults in Guam (Kim et al., 2024)

The 12-item Multidimensional Scale of Perceived Social Support (MSPSS) modified scale assessed participants’ perceived social support across three subscales: Family (*“My family really tries to help me,”*), Friends (*“I can talk about my problems with my friends”*), and Significant Others (*“There is a special person who is around when I am in need”*) (Zimet et al., 1988, 1990). The MSPSS has been psychometrically validated among Pacific Islanders (Kwan et al., 2023). Responses from the MSPSS were scored on a 5-point Likert scale ranging from 1 = ‘Strongly agree’ to 5 = ‘Strongly disagree.’ The mean was computed for each subscale. The measure demonstrated excellent internal consistency for family (α = 0.93), friends (α = 0.93), and significant others (α = 0.95).

Self-stigma was assessed using the 5-item self-esteem subscale of the psychometrically validated Self-Stigma of Mental Illness Scale – Short Version (SSMIS-SF) (Corrigan et al., 2012). Participants responded to five statements (*“I currently respect myself less because I am dangerous”*) using a 5-point Likert scale ranging from 1 = ‘Strongly agree’ to 5 = ‘trongly disagree’. A mean score was calculated from the five items. The measure demonstrated excellent internal consistency (α = 0.91).

### 2.3. Analysis

Descriptive statistics for 213 young, adult Pacific Islanders were generated in SAS 9.4 (SAS Institute Inc, 2013). Differences between participants with versus without severe (Lovibond and Lovibond, 1995) depression (21+), anxiety (15+), or stress (26+) were evaluated utilizing χ2 tests, t-tests, and Cochran-Armitage trend tests. Demographics-adjusted linear regression models calculated unstandardized regression coefficients and 95 % confidence intervals (95 % CI) documenting the association of mental health self-stigma and perceived social support with self-reported levels of depression, anxiety, and stress. The initial models examined the independent effects of stigma and social support. Subsequent models tested an interaction between stigma and each form of social support. Each model incorporated age, ethnicity (Tongan, Samoan, or other Pacific Islander), gender (female, male), education (greater than high school education, high school education or less), employment status (full-time employment, less than full-time employment), and marital status (never married, married) as covariates. All continuous variables were mean-centered prior to model fitting.

Due to the sensitivity of the topic of mental health within the Pacific Islander community, participants were permitted to skip any question within the survey that made them uncomfortable. Although the majority of the questions had less than 5 % of responses missing, the use of list-wise deletion would have eliminated 13.2 % of the observations. Consequently, multivariate imputation by chained equations (van Buuren, 2007) was employed to generate 150 imputed datasets as recommended by a 2-stage analysis (von Hippel, 2020). Parameter estimates from the analysis of imputed datasets were combined according to Rubin’s rules (Rubin, 1987). *P*-values in all models were 2-sided and statistical significance was defined as *P* < 0.05. Statistically significant interactions were visualized utilizing the pick-a-point approach (Bauer and Curran, 2005; Robosa, 1980) and by applying the Johnson-Neyman technique (Johnson and Fay, 1950; Johnson and Neyman, 1936) to identify regions of significance (Hayes and Matthes, 2009).

## 3. Results

The sample (Table 1) was primarily Tongan (50.2 %) and Samoan (32.4 %). Two-third of the participants (66.3 %) were female. The means from each 5-point Likert scale indicated that on average social support was high (≥4) and mental health self-stigma was low (1.9). More than one-fifth of the sample (22.1 %) reported severe (Lovibond and Lovibond, 1995) depression (12.2 %), anxiety (16.8 %), or stress (14.8 %) while 12.7 % reported severe levels of two or more forms of psychological distress. Social support from family was lower and mental health self-stigma was higher among participants with severe depression, anxiety, or stress.

**Fig. 1.**
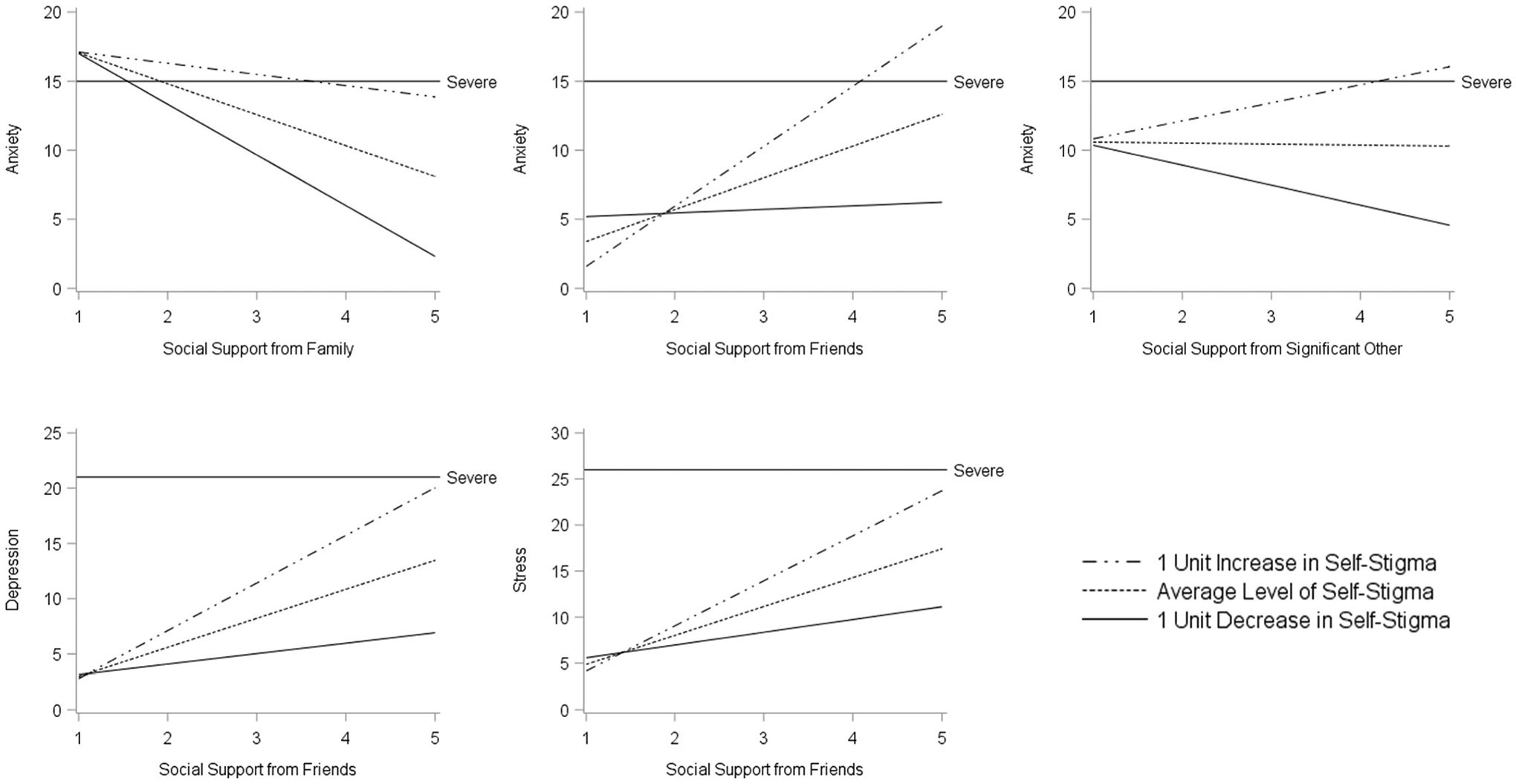
Visual representation of the moderating effect of mental health self-stigma on the relationship between perceived social support and depression, anxiety, or stress among 213 young adult Pacific Islanders. Note: Visualization created by utilizing parameter estimates from linear regression models to generate values for a hypothetical 27-year-old single, female, Tongan with a greater than high school education and full-time employment.

**Fig. 2.**
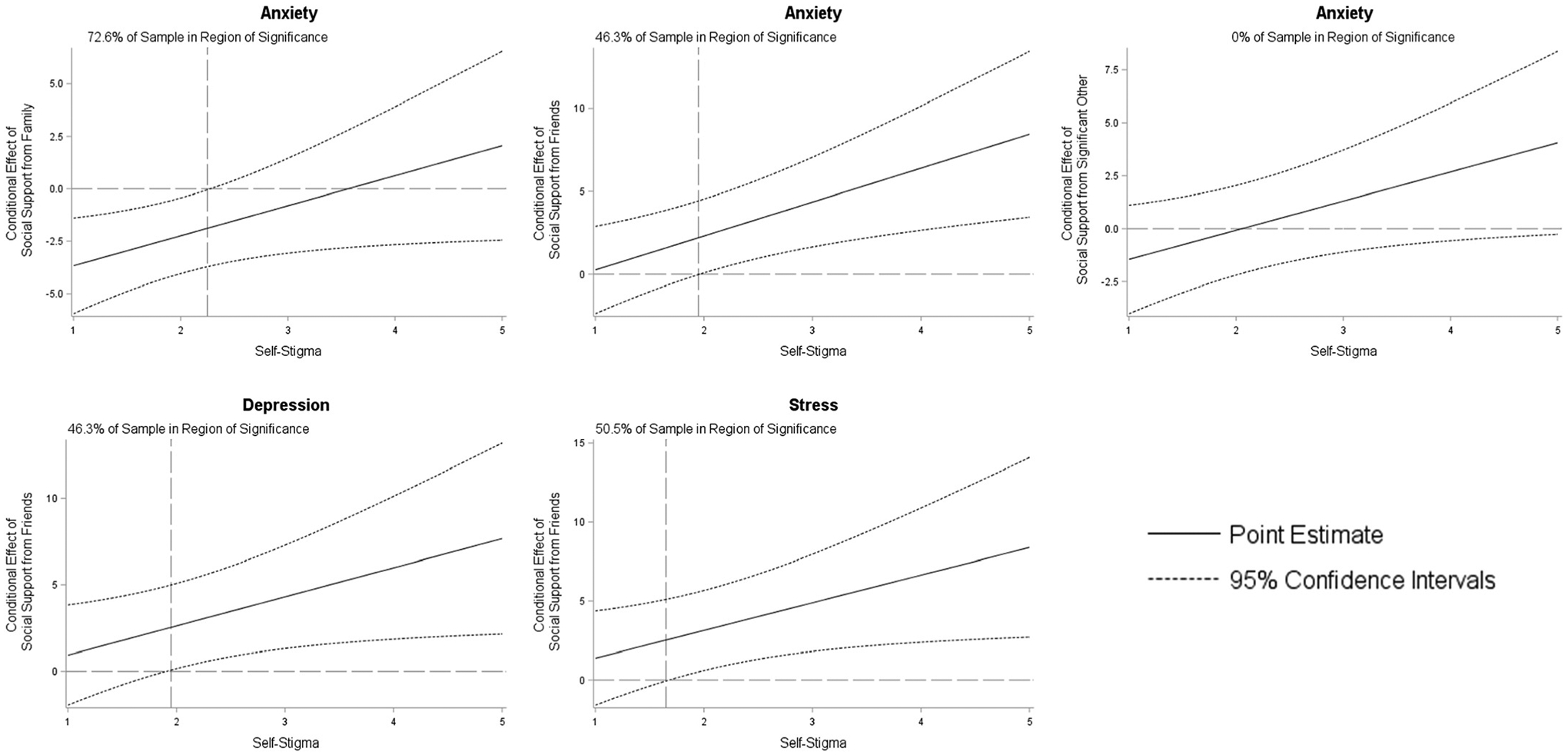
Visual depiction of regions of significance of the conditional effect of perceived social support on depression, anxiety, or stress when moderated by self-stigma among 213 young adult Pacific Islanders. Note: Johnson-Neyman visualization of conditional effect of social support. Dashed lines represent 95 % confidence intervals that can be used to infer statistical significance. When the horizontal line is included in the confidence interval the interaction is not statistically significant. The vertical line denotes the point at which the upper or lower confidence band crosses the zero-line and the interaction becomes statistically significant.

In demographics-adjusted models (Table 2), social support from family was associated with lower levels of depression (*b* = −3.99, 95 % CI = −5.91, −2.08, p < 0.001), anxiety (*b* = −2.29, 95 % CI = −4.05, −0.52, p = 0.01), and stress (*b* = −4.23, 95 % CI = −6.23, −2.24, p < 0.001) while social support from friends was associated higher levels of depression (*b* = 2.48, 95 % CI = 0.09, 4.87, p = 0.04), anxiety (*b* = 2.10, 95 % CI = −0.09, 4.30, p = 0.06), and stress (*b* = 2.96, 95 % CI = 0.46, 5.47, p = 0.02). Mental health self-stigma was independently associated with greater depression (*b* = 4.60, 95 % CI = 3.11, 6.10, p < 0.001), anxiety (*b* = 4.07, 95 % CI = 2.68, 5.45, p < 0.001), and stress (*b* = 4.33, 95 % CI = 2.78, 5.89, p < 0.001) and functioned as a statistically significant moderator that either amplified the deleterious effect of social support from friends on depression, anxiety, or stress or altered the effects of other sources of social support on anxiety (Fig. 1). Region of significance plots (Fig. 2) revealed that the interaction between self-stigma and social support from friends operated in approximately half of the sample. Self-stigma moderated the relationship between social support from family and self-reported anxiety in 72.6 % of the sample. Although the interaction between self-stigma and social support from a significant other was statistically significant, the region of significance suggests that the moderating role of self-stigma only functions at levels beyond those observed in the sample.

## 4. Discussion

This study analyzed the association between mental health self-stigma, perceived levels of social support, and psychological distress (depression, anxiety, stress) among young adult Pacific Islanders living in Southern California. We observed a high prevalence of severe symptoms of depression (12.2 %), anxiety (16.8 %), and stress (14.8 %) in the study sample. Although research on the prevalence of these mental health outcomes are limited among Pacific Islanders, our findings are consistent with estimates from a previous study on heightened psychological distress among Pacific Islanders in the US (Subica et al., 2019b) and Aotearoa New Zealand (Kapeli et al., 2020a). The high prevalence of distress in this study is concerning given only 6.1 % of PIs receive mental health services, the lowest of any racial/ethnic group (SAMHSA, 2020). Moreover, despite our findings revealing a negative association between familial social support and all three mental health outcomes, participants who met thresholds for severe depression, anxiety, or stress also reported higher self-stigma and lower social support from family.

Self-stigma was positively associated with depression, anxiety, and stress, corroborating previous findings that symptoms of psychological distress are elevated in people who exhibit higher self-stigma toward mental illness among Pacific Islander subgroups (Subica et al., 2019a; Allen et al., 2016; Vogel et al., 2007). Study results show that among young adult Pacific Islanders, social support from family was associated with lower levels of depression, anxiety, and stress, indicating that family as a source of support is a protective factor against varying psychological distress similar to Kwan et al. (2023); these findings also parallel other studies wherein family support was found to buffer the negative effects of internalized stigma on mental health and, in some cases, more so than peer or professional support (Allen et al., 2016; Chang, 2008; Lindsey et al., 2010; Li et al., 2020). Further research identified religion, spirituality, and family as primary coping sources for psychological distress among Pacific Islander groups (Allen et al., 2016; Yamada et al., 2019). Although stress is often induced by familial expectations of withholding traditional values in collectivist communities, perhaps established, well-nurtured familial relationships where shared group experiences (e.g. family-related activities, attending events together, strong communication) and idioms of ‘family’ as an extended network of personal connections may contribute to dispersing mental health self-stigma in this US-born centric sample.

Social support from friends was positively associated with psychological distress and was amplified by self-stigma. The moderating effect of self-stigma on social support from friends could be explained by feelings of inferiority and concerns about how one is perceived within one’s own social networks (Vogel et al., 2007). As suggested by other researchers (Allen et al., 2016; Kalibatseva, 2015; Ran et al., 2021), one explanation for this is that the cultural processes one is exposed to can foster public acceptance of mental illness as taboo, thus breeding self-stigma if one agrees, whether consciously or subconsciously, with these processes. Similarly, it is possible that the peer social supports that individuals in the current sample turn to also hold stigmatizing views on mental health. In such cases, self-stigma and psychological distress are likely to worsen in the face of low peer support, especially superficial connections or among friends who do not share similar cultural, value systems. Young adults inherently undergo multi-dimensional developmental transitions that occur simultaneously through life events (e.g. career, role shifts, marriage) in environmental settings (e.g. school, work, households), between interpersonal bonds (e.g. family, friends, romantic relationships), and within internal processes (e.g. independence, facing new/challenging responsibilities and experiences, vulnerability, instability) (Spencer et al., 2023). Interdependent and intersecting are the components of collectivistic Pacific Islander culture that adds various complex layers of push-pull factors such as connection to tradition, identity exploration and expression, discrimination, and erasure that influence mental health outcomes (Kapeli et al., 2020b; Spencer et al., 2023; Yee et al., 2007). Interventions that focus on increasing mental health awareness during transitional years (e.g. adolescence to young adulthood) that take into account cultural definitions of connectedness and references (e.g. sociopolitical histories, language, religious/spiritual values), building trust among diverse groups of peers to cope with mental distress, and educating young adult Pacific Islanders on identifying and combatting self-stigma, such as structural stressors and poor coping mechanisms within their social circles, are viable options that could help counter the negative influence of peer social support (Kapeli et al., 2020b; Vogel et al., 2007; Lannin et al., 2016). Still, additional studies are needed to understand the different pathways by which peer social support can influence mental health and stigma processes in young adult Pacific Islanders; an additional nuance is to specifically assess who they normally define as friends versus family – which often includes close friends and extended members; this differs from Western relational definition of nuclear family members only (e.g. siblings, parents, grandparents) (Kapeli et al., 2020b).

Stigma is considered one of the primary barriers to seeking mental health services and adhering to treatment (Corrigan, 2004, 2007; Komiya et al., 2000; Subica et al., 2019; Adewuya et al., 2011; Schnyder et al., 2017; Carrara and Ventura, 2018; Vogel et al., 2007), especially among people of color (Allen et al., 2016). Heightened self-stigma prompts individuals to suppress distress and makes them more apprehensive to seeking mental health resources for fear of community chastisement, invalidation from others, appearing weak or dangerous, and loss of important relationships (Subica et al., 2019; Lannin et al., 2016; Yamada et al., 2019; Phan, 2020). Therefore, interventions to reduce both public stigma and self-stigma among young adult Pacific Islanders are needed; indeed, studies have shown that internalized stigma decreases with anti-stigma interventions (Livingston and Boyd, 2010). Culturally-tailored approaches, such as those recommended by Yamada et al. (2019), also warrant consideration. In their seminal study on community providers’ perspectives of the mental health challenges facing Samoan Americans living in Southern California, they revealed that providers favored the use of existing resources to increase mental health knowledge, reduce stigma, and increase service utilization among this population. Applicable to other Pacific Islander populations, such resources may include spiritual/religious spaces, media outlets (e.g., radio, newspapers), and other group activities (e.g. sports, performing arts) frequently used by Pacific Islanders, and partnerships with community-based organizations that serve Pacific Islanders (Yamada et al., 2019). New resources are also imperative, such as training culturally responsive health care providers who are receptive to the mental health needs of Pacific Islander patients (Yamada et al., 2019), utilizing Pacific Islander data and paradigms for emphasizing health importance, and collaborating with Pacific Islander mental health professionals.

In addition to anti-stigma efforts, we must not overlook the ongoing mental health crisis unfolding as a result of the COVID-19 pandemic, like discrimination and isolation issues. Limited data from 2020 to 2021 indicated that Native Hawaiian and Pacific Islanders had the highest COVID-19 infection, hospitalization, and mortality rates of any racial/ethnic group in the US (Cha et al., 2022; Subica et al., 2022), likely compounding existing healthcare and mental health disparities in these populations. With mental health services disrupted and a more than 25 % increase in depression and anxiety disorders globally (World Health Organization, 2022), the pandemic highlighted persistent challenges to the mental health needs of Pacific Islanders (National Urban League, 2022). For example, Subica et al. (2022) found that 27 % and 19 % of Native Hawaiians and Pacific Islanders met diagnostic thresholds for depression and anxiety disorders, respectively, and 60 % of those who reported needing past-year mental health treatment avoided or delayed treatment. Another study that surveyed Native Hawaiian and Pacific Islanders across five states in 2021 also found that 16 % had a close family member die of COVID-19 and that those who experienced such loss were more likely to have heightened psychological distress (Subica et al., 2023). Similar rates were found with a national COVID-19 Communities of Color Needs Assessment for Native Hawaiian and Pacific Islanders (National Urban League, 2022) where 38 % of their respondents reported either depression or anxiety symptoms, with most depression/anxiety reports from those of annual household incomes less than $25,000 (40 %) and among 18–24 year olds (46 %). As we approach a post-COVID-19 era, we are faced not only with compounded healthcare and mental health challenges, but also with new opportunities to effectively improve the influence of social support as a catalyst for improving the mental health of Pacific Islanders, specifically leveraging social circles for seeking healthcare/intervention while partnering with Pacific Islander-led community-based organizations to improve resources, data collection approaches, and evidence-based interventions to reduce stigma (i.e. cultural, self) while increasing mental health literacy. Because of compounding psychosocial health issues underscored by the pandemic, there is a strong commitment in the Pacific Islander community to continue providing referrals for culturally competent wellness providers, combating stigma through workshops and talk story groups, and advocating for policies that provide equitable access for healthcare and mental resources for Pacific Islanders (MALO, 2025; So Cal Pacific Islander Community Response Team, 2025).

The present study presents several limitations to data results and interpretations. Our use of a cross-sectional design does not allow for inferences on causality or on the directionality between self-reported self-stigma, social support, and psychological distress. Moreover, some of these measures, such as the short version of the Self-Stigma of Mental Illness Scale, have not been validated among young, adult Pacific Islanders and may not capture the full meaning of these constructs. Each measure also relied on self-report which is subject to response bias. Social support, for example, is an obscure construct with many definitions that have broadened overtime, like include online platforms (e.g., social networking sites) (Trepte and Scharkow, 2016; Verduyn et al., 2017). This is especially relevant to the young adult population given those aged 18 to 29 make up 84 % of social media users (Auxier and Anderson, 2021). Further research would benefit from expanding measures of social support (Nick et al., 2018) to include the size of one’s online social networks and the frequency of use of these networks for social support, in addition to broadening the cultural assessment of social support and wellbeing among collectivistic communities, like Pacific Islanders (Kapeli et al., 2020b).

Our study focused primarily on Pacific Islanders of Samoan (32.4 %) and Tongan (50.2 %) ethnicities living in the greater Los Angeles area. Other nuances may impact our study’s generalizability to other Pacific Islanders living throughout the US such as geographic location, migration time, generational household/peer differences, social activity networks, etc. Although 82.9 % of participants were US born, we acknowledge the differences in the types of support and intensity of stigma provided by family/peers, some of whom may be non-immigrant, uphold traditional beliefs about mental illness, and/or unaware/weary of US mental health services or treatment. In addition, two-thirds of our sample was female, thus obscuring any meaningful gender differences in our analysis. Although our data aligns with literature where supportive familial relationships were inversely related to depression among females and that positive levels of social support buffered against psychological distress among Pacific Islander young adult women (Kapeli et al., 2020b), additional research is needed to account for potential sex-and gender-specific differences in mental health outcomes and social support perceptions among this population. A longitudinal cohort study may also better assess the dynamic mental health landscape of Pacific Islanders post pandemic and during critical life stages, as it relates to their changing social environment. Future studies focused on communities of color or indigenous groups may also benefit to include or analyze diverse Pacific Islander subethnic groups to further contribute to the growing literature on the unique or similar mental health experiences of Pacific Islanders across the diaspora, locally and abroad.

The role of social support in mental health attitudes and outcomes is complex within diverse populations, one that complicates the effects of stigma on mental health and is often neglected in mental health interventions as a cultural component for communities of color and indigenous groups. Our findings illuminate this complex relationship between self-stigma and social support among young adult Pacific Islanders, an at risk group for both high-levels of psychological distress and suicidality. Incorporating protective community-level factors (i.e., family, church groups, special interest communities) in anti-stigma interventions and increasing education on the role of family and peer support in self-stigma are potential avenues toward mitigating stigma, increasing culturally-appropriate resources for mental health service utilization, and, in turn, improving mental health outcomes among this population. The benefits of exploring the types and contexts of social support will remain increasingly relevant in culturally tailored interventions as we move forward to improve the mental health of young adult Pacific Islanders and over the life course.

## Figures and Tables

**Table 1:** Characteristics of 213 young adult Pacific Islanders assessed between 2018 and 2019.

			Depression			Anxiety			Stress		
		All	<21	21+		<15	15+		<26	26+	
	N	(N = 213)	(N = 166)	(N = 23)	P	(N = 158)	(N = 32)	P	(N = 161)	(N = 28)	P
Age, mean (SD), y	213	27.0 (5.3)	27.2 (5.3)	26.2 (5.4)	0.42	27.0 (5.3)	27.6 (5.5)	0.57	27.2 (5.3)	26.2 (5.1)	0.36
Ethnicity, No. (%)											
Samoan	213	69 (32.4)	52 (31.3)	7 (30.4)	0.93	49 (31.0)	10 (31.3)	0.98	50 (31.1)	9 (32.1)	0.91
Tongan		107 (50.2)	86 (51.8)	12 (52.2)	0.97	83 (52.5)	16 (50.0)	0.79	88 (54.7)	10 (35.7)	0.06
Other Pacific Islander		37 (17.4)	28 (16.9)	4 (17.4)	0.95	26 (16.5)	6 (18.8)	0.75	23 (14.3)	9 (32.1)	0.02
Female Gender, No. (%)	208	138 (66.3)	107 (66.0)	16 (69.6)	0.74	101 (65.6)	23 (71.9)	0.49	104 (66.2)	19 (67.9)	0.87
Greater than High School Education, No. (%)	211	141 (66.8)	111 (66.9)	15 (65.2)	0.87	103 (65.2)	24 (75.0)	0.28	104 (64.6)	22 (78.6)	0.15
Full-Time Employment, No. (%)	209	117 (56.0)	91 (54.8)	14 (60.9)	0.58	87 (55.4)	18 (56.3)	0.93	88 (54.7)	17 (60.7)	0.55
Single, Never Married, No. (%)	213	145 (68.1)	110 (66.3)	17 (73.9)	0.46	104 (65.8)	24 (75.0)	0.31	106 (65.8)	21 (75.0)	0.34
Mental Health Self-Stigma, mean (SD)	190	1.9 (0.9)	1.7 (0.8)	2.7 (1.0)	<0.001	1.7 (0.9)	2.4 (1.0)	<0.001	1.7 (0.8)	2.5 (1.1)	<0.001
Social Support, mean (SD)											
Family	194	4.0 (1.0)	4.1 (1.0)	3.4 (1.3)	0.001	4.1 (1.0)	3.6 (1.3)	0.01	4.1 (1.0)	3.5 (1.2)	0.004
Friends	194	4.2 (0.9)	4.2 (0.9)	4.0 (1.1)	0.16	4.2 (0.9)	4.1 (1.1)	0.64	4.2 (0.9)	4.3 (0.9)	0.82
Significant Other	194	4.2 (1.0)	4.2 (1.0)	3.9 (0.9)	0.11	4.2 (1.0)	4.1 (1.0)	0.52	4.2 (1.0)	4.1 (0.9)	0.42
DASS 21: Depression Score, No. (%)											
Normal (0–9)	189	135 (71.4)	135 (81.3)	0 (0.0)	<0.001	131 (83.4)	4 (12.5)	<0.001	131 (81.4)	4 (14.3)	<0.001
Mild (10–13)		11 (5.8)	11 (6.6)	0 (0.0)		8 (5.1)	3 (9.4)		9 (5.6)	2 (7.1)	
Moderate (14–20)		20 (10.6)	20 (12.0)	0 (0.0)		16 (10.2)	4 (12.5)		16 (9.9)	4 (14.3)	
Severe (21–27)		7 (3.7)	0 (0.0)	7 (30.4)		1 (0.6)	6 (18.8)		4 (2.5)	3 (10.7)	
Extremely Severe (28+)		16 (8.5)	0 (0.0)	16 (69.6)		1 (0.6)	15 (46.9)		1 (0.6)	15 (53.6)	
DASS 21: Anxiety Score, No. (%)											
Normal (0–7)	190	117 (61.6)	116 (69.9)	1 (4.3)	<0.001	117 (74.1)	0 (0.0)	<0.001	115 (71.4)	2 (7.1)	<0.001
Mild (8–9)		9 (4.7)	9 (5.4)	0 (0.0)		9 (5.7)	0 (0.0)		9 (5.6)	0 (0.0)	
Moderate (10–14)		32 (16.8)	30 (18.1)	1 (4.3)		32 (20.3)	0 (0.0)		24 (14.9)	7 (25.0)	
Severe (15–19)		7 (3.7)	3 (1.8)	4 (17.4)		0 (0.0)	7 (21.9)		6 (3.7)	1 (3.6)	
Extremely Severe (20+)		25 (13.2)	8 (4.8)	17 (73.9)		0 (0.0)	25 (78.1)		7 (4.3)	18 (64.3)	
DASS 21: Stress Score, No. (%)											
Normal (0–14)	189	132 (69.8)	132 (79.5)	0 (0.0)	<0.001	129 (82.2)	3 (9.4)	<0.001	132 (82.0)	0 (0.0)	<0.001
Mild (15–18)		12 (6.3)	11 (6.6)	1 (4.3)		9 (5.7)	3 (9.4)		12 (7.5)	0 (0.0)	
Moderate (19–25)		17 (9.0)	13 (7.8)	4 (17.4)		10 (6.4)	7 (21.9)		17 (10.6)	0 (0.0)	
Severe (26–33)		19 (10.1)	9 (5.4)	10 (43.5)		8 (5.1)	11 (34.4)		0 (0.0)	19 (67.9)	
Extremely Severe (34+)		9 (4.8)	1 (0.6)	8 (34.8)		1 (0.6)	8 (25.0)		0 (0.0)	9 (32.1)	

Note: Participants were permitted to skip survey questions they did not want to answer. Consequently, the number of complete cases varied for each measure. The analytic sample was stratified by thresholds for severe (Lovibond and Lovibond, 1995) depression (21+), anxiety (15+), or stress (26+). Univariate differences between subgroups were assessed using χ2 tests, t tests, and Cochran-Armitage trend tests as appropriate.

**Table 2:** Demographics-adjusted linear regression models examining the effect of mental health self-stigma and perceived social support on depression, anxiety, and stress among 213 young adult Pacific Islanders.

	No Interaction		Interaction with		Interaction with		Interaction with Support	
			Family Support		Friend Support		from Significant Other	
	*b (95 % CI)*	*p*	*b (95 % CI)*	*p*	*b (95 % CI)*	*p*	*b (95 % CI)*	*p*
**Outcome: Depression**								
Mental Health Self-Stigma	4.60 (3.11, 6.10)	<0.001	4.87 (3.32, 6.43)	<0.001	4.88 (3.33, 6.43)	<0.001	4.93 (3.37, 6.48)	<0.001
Social Support from Family	−3.99 (−5.91, −2.08)	<0.001	−4.17 (−6.16, −2.18)	<0.001	−4.06 (−6.04, −2.08)	<0.001	−3.98 (−5.97, −1.99)	<0.001
Social Support from Friends	2.48 (0.09, 4.87)	0.04	2.55 (0.08, 5.03)	0.04	2.62 (0.16, 5.08)	0.04	2.46 (−0.00, 4.92)	0.05
Social Support from Significant Other	0.62 (−1.63, 2.86)	0.59	0.73 (−1.58, 3.04)	0.54	0.49 (−1.81, 2.80)	0.68	0.53 (−1.77, 2.84)	0.65
Self-Stigma X Social Support			1.04 (−0.48, 2.55)	0.18	1.68 (0.07, 3.29)	0.04	1.36 (−0.06, 2.78)	0.06
**Outcome: Anxiety**								
Mental Health Self-Stigma	4.07 (2.68, 5.45)	<0.001	4.35 (2.93, 5.76)	<0.001	4.33 (2.93, 5.74)	<0.001	4.36 (2.95, 5.78)	<0.001
Social Support from Family	−2.29 (−4.05, −0.52)	0.01	−2.24 (−4.05, −0.43)	0.02	−2.11 (−3.90, −0.31)	0.02	−2.05 (−3.86, −0.23)	0.03
Social Support from Friends	2.10 (−0.09, 4.30)	0.06	2.22 (−0.03, 4.47)	0.05	2.30 (0.08, 4.53)	0.04	2.12 (−0.12, 4.37)	0.06
Social Support from Significant Other	0.19 (−1.86, 2.23)	0.86	0.15 (−1.95, 2.25)	0.89	−0.15 (−2.24, 1.94)	0.89	−0.07 (−2.18, 2.03)	0.95
Self-Stigma X Social Support			1.43 (0.05, 2.81)	0.04	2.05 (0.58, 3.51)	0.006	1.38 (0.08, 2.67)	0.04
**Outcome: Stress**								
Mental Health Self-Stigma	4.33 (2.78, 5.89)	<0.001	4.56 (2.96, 6.16)	<0.001	4.55 (2.95, 6.14)	<0.001	4.57 (2.96, 6.18)	<0.001
Social Support from Family	−4.23 (−6.23, −2.24)	<0.001	−4.31 (−6.36, −2.26)	<0.001	−4.20 (−6.24, −2.15)	<0.001	−4.15 (−6.21, −2.09)	<0.001
Social Support from Friends	2.96 (0.46, 5.47)	0.02	3.06 (0.52, 5.61)	0.02	3.13 (0.60, 5.67)	0.02	2.98 (0.43, 5.53)	0.02
Social Support from Significant Other	0.27 (−2.06, 2.59)	0.82	0.31 (−2.07, 2.68)	0.80	0.05 (−2.32, 2.42)	0.97	0.11 (−2.27, 2.50)	0.92
Self-Stigma X Social Support			1.27 (−0.29, 2.83)	0.11	1.75 (0.09, 3.41)	0.04	1.17 (−0.30, 2.63)	0.12

Note: Unstandardized parameter estimates generated from linear regression models that incorporated age, ethnicity, gender, education, employment status, and marital status as covariates. Multiple imputation by chained equations was employed to account for missing responses.
